# Predicting Covid-19 preventive behaviors based on constructs of health belief model

**DOI:** 10.1017/S1463423622000743

**Published:** 2023-02-10

**Authors:** Azadeh Heydari, Parvaneh Isfahani, Somayeh Bagheri

**Affiliations:** 1 Department of Public Health, School of Public Health, Zabol University of Medical Sciences, Zabol, Iran; 2 Department of Health Services Management, School of Public Health, Zabol University of Medical Sciences, Zabol, Iran

**Keywords:** awareness, Covid-19, health belief model, preventive behaviors

## Abstract

**Introduction and objective:**

The prevalence of Covid-19 has become a clinical threat worldwide. However, knowledge about this new virus is limited. Therefore, this study was conducted to determine the preventive behaviors of Covid-19 based on the constructs of health belief model (HBM) in the clients of health centers in Zabol, Iran.

**Methods:**

This descriptive–analytical (cross-sectional) study was performed on 160 people referring to health centers in Zabol by the available method. A researcher-made questionnaire was used to collect data. Data were analyzed using SPSS20 software and appropriate statistical tests.

**Results:**

A positive and significant correlation was observed between preventive behaviors of Covid-19 and perceived benefit constructs (*r* = 0.29, *P* = 0.0001) and self-efficacy (*r* = 0.39, *P* = 0.0001). HBM constructs were able to predict about 36% of the variance of Covid-19 disease preventive behaviors, with self-efficacy (*B* = 0.134) being the strongest predictor.

**Conclusion:**

Since no specific and definitive treatment for Corona has been found yet, taking preventive measures can be the best way to prevent the spread of this disease in the community. Therefore, this can be achieved by raising awareness and preventive behaviors through health education.

## Introduction

Seasonal flu epidemic is one of the serious public health concerns that annually leads to respiratory diseases in tens of millions of people and death of 250–500 thousand people around the world (World Health Organization, 2014). In addition to seasonal flu, there are new types of the flu virus for which there is no vaccine, and human-to-human transmission can lead to pandemics and the death of millions. By quickly identifying the flu and responding quickly, its negative effects can be significantly reduced. On December 8, 2019, Chinese officials reported a new type of coronavirus to the World Health Organization that caused an infectious disease with symptoms of fever, cough and shortness of breath. The disease originated in the seafood market in Wuhan, China (Huang *et al.*, 2020).

The incubation period of the disease varies between 2 and 14 days. On January 6, 2020, the World Health Organization named the disease Covid-19 (Wang *et al.*, 2020). Covid-19 is an infectious disease caused by a new coronavirus. Most people infected with Covid-19 virus experience a mild to moderate respiratory illness and recover without special treatment (World Health Organization, 2020a). However, some people develop Severe Acute Respiratory Syndrome Coronavirus, which can lead to death. The incubation period of severe acute respiratory syndrome coronavirus (SARS-CoV-2) has been reported differently. For example, the US Centers for Disease Control and Prevention reported an incubation period of 2–14 days, the World Health Organization between 2 and 10 days, and the Chinese National Health Commission between 10 and 14 days (CDC, 2020; Coronavirus, 2020, World Health Organization, 2020b).

The prevalence of this disease in Iran was officially confirmed on February 18, 2020. According to the public relations of the Ministry of Health, Treatment and Medical Education of Iran, up to April 12, 2021, 2 070 000 patients with Covid-19 have been identified in the country, of which 64 490 have died and 1 710 974 have recovered (Coronavirus, 2021a). According to the official statistics provided by Zahedan University of Medical Sciences, a total of 64 people with Covid-19 were admitted to hospitals in Sistan and Baluchestan province on April 1, 2021, of which eight people were related to hospitals affiliated with Zabol University of Medical Sciences (Coronavirus, 2021b).

Measures such as training, awareness raising, attitude, and taking preventive action to protect against Covid-19 can be important strategies to prevent Covid-19 (Khazaee-Pool *et al*., 2020). Therefore, choosing an appropriate model for teaching preventive behaviors is the first step in health planning. One of the appropriate models for teaching disease prevention behaviors is the health belief model (HBM). This model predicts the relationship between health beliefs and behavior and provides a useful theoretical framework for research into the recognition of a wide range of behavioral determinants (Sabet Rouhani *et al*., 2005).

According to this model, in order to apply preventive behaviors, a person must first feel threatened by the causative problem of Covid-19 (perceived sensitivity), then understand the severity of its complications (perceived severity), with positive symptoms received from the environment (action guide), believes that the Covid-19 prevention program is feasible (perceived benefits) and finds action deterrents less costly than its benefits (perceived barriers) in order to take preventive measures against the Covid-19. On the other hand, positive judgment about person’s abilities to adopt preventive behaviors against Covid-19 (perceived self-efficacy) is also an accelerating force that leads to the need to adopt preventive behaviors against the disease (Khazaee-Pool *et al*., 2020). Therefore, this study was performed by determining the preventive behaviors against Covid-19 based on the constructs of the HBM in the clients of health centers in Zabol.

## Method

This descriptive–analytical cross-sectional study was conducted in 2020. Available sampling method was among patients referred to urban health centers of which 160 people participated in the study. Inclusion criteria included the willingness to participate in the study. Exclusion criteria were unwillingness to continue cooperation while answering an incomplete questionnaire. The data collection tool was a researcher-made questionnaire consisting of four parts. The first part included demographic information, the second part included questions related to awareness, and the third part included questions related to HBM constructs. The first part of the questionnaire included demographic information (7 items). The second part included awareness questions consisted of 10 questions with a score of 0 and 1, in which 1 point was assigned to the correct answer and 0 point to the wrong answers. In addition, people who scored between 0 and 33.3% in each section had poor awareness, people who scored between 33.3 and 66.7% had moderate knowledge, and then people who scored above 66.7% had a good level of awareness.

The third part includes the constructs of the HBM after perceived sensitivity (3 questions), perceived severity (4 questions), perceived benefits (4 questions), perceived barriers (4 questions), action guide (1 question), and self-efficacy (4 questions). Scoring was based on 3-point Likert (I agree, I have no opinion, and I disagree) and from 1 to 3. Perceived barriers questions were scored in reverse. The action guide included one multiple-choice question, including advice from healthcare staff/radio and television, and more. The preventive behaviors dimension consisted of five questions and an achievable score ranged from 0 to 10. The right behavior was given 2 points, and the wrong behavior was not given points. In addition, the mean of such preventive behaviors was 0 to 3 points for poor behavior, 4–6 for moderate behavior, and 7–10 for good behavior.

In order to determine the validity of the content, the opinions of several health education specialists and physicians were used. For reliability, a pilot study was performed on 16 patients referred to urban health centers of Zabol and Cronbach’s alpha coefficient was above 0.70. Data analysis was performed using frequency, mean, standard deviation, Pearson correlation coefficient, ANOVA, *t*-test, and linear regression based on SPSS20 software at a significance level of 0.05. Shapiro–Wilk pretest was used to check the normality of the samples. Obtaining an ethics code from the Research Deputy of Zabol University of Medical Sciences (IR.ZBMU.REC.1399.057), obtaining informed consent, the participant’s freedom to participate in the study, maintaining the confidentiality of the participant’s personal information and the impartiality of researchers in all stages of collection, data analysis, and reporting were among the ethical considerations observed in this study.

## Results

The mean age of participants was 12.33 ± 34.51 years. Most of the participants were women (58.8%), housewives (53.2%), had incomes below 2 million Tomans (70.6%) and had undergraduate education (46.3%). About 94.4% of participants had no history of Corona (Table 1).

**Table 1.** Frequency distribution of demographic specifications in the study population

Variable		Percent
Gender	Female	58.7
	Male	41.3
Job	Housewife	53.2
	Worker	8.1
	Student	6.3
	Unemployed	5.6
	Self-employed	8.1
	Farmer	8.1
	Employee	8.1
	Retired	2.5
Monthly income	Under 2 million Tomans	70.6
	Between 2–4 million Tomans	18.1
	More than 4 million Tomans	11.3
Education	Undergraduate	46.3
	Diploma	28.7
	Graduate	25
History of Corona	Yes	5.6
	No	94.4
History of family members’ Corona	Yes	10
	No	90

Among the preventive behaviors against Covid-19 disease, “using a mask when leaving home” (98.1%) had the highest frequency, respectively. “Immediate change of clothes when returning home” (52.5%) also had the lowest frequency (Table 2). About 87.5% of the participants stated that if they had Corona symptoms, they would stay at home and self-medicate.

**Table 2.** Frequency distribution of answers to questions of preventive behaviors against Covid-19 disease in the study population

	Yes	No
Behavior	*n* (%)	*n* (%)
Do you use mask when leaving home?	157 (98.1)	3 (1.9)
Do you use gloves when leaving home?	125 (78.1)	35 (21.9)
Do you wash your hands and face at least for 20 s with soap and water?	154 (96.2)	6 (3.8)
Do you immediately change your clothes while returning home?	84 (52.5)	76 (47.5)

According to Table 3, the most important source of integrated information on education in television was personnel of the Ministry of Health and social networks (41.9%), then television (40.6%), employees of the Ministry of Health (8.1%), and social networks (7.5%).

**Table 3.** Reliable sources of information about Covid-19 in the study population

The most important reliable sources of information about Covid-19	Percent (%)
Television	40.6
Personnel of the Ministry of Health	8.1
Social networks	7.5
Doctor	1.9
Integrated	41.9

The mean score of awareness (15.2 ± 28.7) and perceived benefits (11.1 ± 39.4) was higher than other constructs. About 75.6% of people with awareness, 90% of people with perceived sensitivity, 85% of people with perceived severity, 93.1% of people with perceived benefits, 58.1% of people with perceived barriers, about 95.6% of people with perceived self-efficacy and about 54.4% of people with preventive behaviors against Covid-19 were in good condition (Table 4).

**Table 4.** Mean and standard deviation and leveling of constructs of health belief model and corona preventive behaviors in the study population

Variable	SD ± Mean	Leveling		
		Poor (%)	Average (%)	Good (%)
Awareness	15.2 ± 28.7	0	24.4	75.6
Perceived sensitivity	8.1 ± 45	0	10	90
Perceived severity	10.1 ± 6.8	1.9	13.1	85
Perceived benefits	11.1 ± 39.4	0	6.9	93.1
Perceived barriers	8.2 ± 6.9	15.6	26.3	58.1
Perceived self-efficacy	10.1 ± 92.33	0	4.4	95.6
Preventive behaviors against Covid-19	7.1 ± 12.84	1.9	43.8	54.4

The results of Pearson correlation test showed that there was a significant relationship between awareness with perceived sensitivity (*r* = -0.16, *P* = 0.03) and perceived barriers (*r* = 0.27, *P* = 0.0001). Also, the correlation test showed a significant relationship between preventive behaviors with perceived benefits (*r* = 0.29, *P* = 0.0001) and self-efficacy (*r* = 0.39, *P* = 0.0001). On the other hand, there was correlation between the constructs of perceived sensitivity and severity (*r* = 0.31, *P* = 0.0001), perceived benefits and severity (*r* = 0.30, *P* = 0.0001), perceived barriers and severity (*r* = 0.25, *P* = 0.001), and perceived barriers and benefits (*r* = 0.40, *P* = 0.0001).

There was a relationship between perceived benefits, gender, and perceived self-efficacy with preventive behaviors. The strongest correlation was observed between perceived self-efficacy constructs and preventive behaviors. Linear multiple regression analysis was used to evaluate the prediction of preventive behaviors against Covid-19 by HBM constructs and other variables. The studied variables predicted approximately 36% of the variance of Covid-19 preventive behavior (Table 5).

**Table 5.** Prediction regression coefficient of factors related to preventive behaviors against Covid-19 in the study population

Independent variables	*B*	SD	Standardized cofficients (SB)	*t*	*P*	*R* ^2^
Constant	0.607	0.656	–	0.934	0.035	2 = 0.363 *R* ^2^ adjusted = 0.301
Age	-0.004	0.003	-0.039	-1.170	0.244	
Gender	-0.323	0.110	-0.300	-2.97	0.003	
Job	-0.027	0.030	-0.122	-0.892	0.374	
Income	0.132	0.082	0.169	1.620	0.107	
Education	0.020	0.065	0.033	0.336	0.737	
History of Corona	-0.172	0.175	-0.073	-0.976	0.331	
History of family members’ Corona	-0.119	0.136	-0.068	-0.886	0.377	
Awareness	0.018	0.106	0.017	0.205	0.838	
Perceived sensitivity	0.010	0.023	0.039	0.474	0.636	
Perceived severity	-0.006	0.038	-0.012	-0.154	0.878	
Perceived benefits	0.112	0.031	0.296	3.644	0.0001	
Perceived barriers	0.002	0.016	0.009	0.112	0.911	
Perceived self-efficacy	0.134	0.029	0.336	4.677	0.0001	

The relationship between each of the demographic variables and each of the main variables of the research is given in Table 6. There was a significant relationship between awareness and age, gender, Person’s History of Corona, and job. There was a significant relationship between perceived sensitivity and education. Perceived severity was significantly associated with gender, education, and job. On the other hand, perceived benefits were significantly related to gender. There was a relationship between perceived barriers and gender, education, monthly income, and job. There was a relationship between self-efficacy between Family’s History of Corona, gender, and job. There was a significant relationship between preventive behaviors and age, gender, monthly income, and job.

**Table 6.** Relationship between each demographic variables and main variables

Variables	Gender^**^		Person’s history of Corona^**^		Family’s history of Corona^**^		Education^*^			Monthly income^*^			Job^*^								Age^*^
	Female	Male	Yes	No	Yes	No	Undergraduate	Diploma	Graduate	<2	2–4	>4	Housewife	Worker	Student	Unemployed	Self-employed	Farmer	Employee	Retired	
Awareness	2.42 ± 14.76	2.92 ± 16.01	17.77 ± 1.30	15.13 ± 2.69	15.75 ± 2.72	15.22 ± 2.70	14.93 ± 2.69	15.50 ± 2.72	15.67 ± 2.68	14.94 ± 2.71	16.62 ± 2.28	15.22 ± 2.71	14.50 ± 2.43	13.93 ± 4.09	16.90 ± 1.91	16.55 ± 1.13	15.00 ± 4.02	16.76 ± 1.58	14.00 ± 0.00	15.28 ± 2.70	-0.331
*P*-value		0.004		0.004		0.466			0.305			0.11								0.000	0.000
Perceived sensitivity	8.41 ± 1.08	8.50 ± 1.09	8.77 ± 0.66	8.43 ± 1.10	8.56 ± 1.03	8.43 ± 1.09	8.12 ± 1.30	8.73 ± 0.681	8.72 ± 0.846	8.34 ± 1.17	8.65 ± 0.936	8.77 ± 0.427	8.26 ± 1.17	8.07 ± 1.25	8.30 ± 1.63	9.00 ± 0.00	8.76 ± 0.438	8.38 ± 1.50	8.96 ± 0.196	8.00 ± 0.00	-0.131
*P*-value		0.627		0.353		0.664			0.002			0.155								0.051	0.099
Perceived severity	10.29 ± 2.00	11.21 ± 1.53	11.00 ± 2.00	10.65 ± 1.87	10.62 ± 2.12	10.68 ± 1.85	10.13 ± 2.19	11.39 ± 1.21	11.57 ± 0.930	10.69 ± 1.86	10.96 ± 2.04	10.11 ± 1.64	10.18 ± 2.08	11.84 ± 0.55	11.30 ± 1.25	11.55 ± 0.726	10.92 ± 1.49	11.84 ± 0.5	10.46 ± 2.21	9 ± 0.00	0.025
*P*-value		0.002		0.595		0.911			0.001			0.315								0.002	0.750
Perceived benefits	11.20 ± 1.68	11.66 ± 0.810	12.00 ± 0.00	11.35 ± 1.43	10.87 ± 1.66	11.45 ± 1.36	11.12 ± 1.78	11.67 ± 0.920	11.57 ± 0.930	11.29 ± 1.61	11.82 ± 0.53	11.33 ± 0.68	11.11 ± 1.81	11.30 ± 0.751	11.00 ± 2.10	12.00 ± 0.00	11.69 ± 0.48	12.00 ± 0.00	11.76 ± 0.58	11.00 ± 0.00	-0.040
*P*-value		0.039		0.184		0.120			0.071			0.184								0.155	0.616
Perceived barriers	7.92 ± 2.96	9.72 ± 2.45	10.33 ± 1.93	8.56 ± 2.92	8.62 ± 2.41	8.67 ± 2.95	7.89 ± 3.00	8.21 ± 2.95	10.62 ± 1.42	8.12 ± 2.96	9.55 ± 2.54	10.66 ± 1.64	7.47 ± 2.99	7.23 ± 3.44	9.20 ± 2.20	8.88 ± 2.93	10.00 ± 1.52	10.69 ± 0.63	10.23 ± 1.94	12.00 ± 0.00	-0.143
*P*-value		0.000		0.076		0.950			0.000			0.000								0.000	0.070
Self-efficacy	11.14 ± 1.34	10.62 ± 1.28	10.88 ± 1.45	10.93 ± 1.33	10.12 ± 1.82	11.02 ± 1.25	11.14 ± 1.16	10.80 ± 1.45	10.67 ± 1.47	10.97 ± 1.29	11.13 ± 1.12	10.33 ± 1.78	11.27 ± 1.18	10.84 ± 1.14	11.00 ± 1.76	10.00 ± 1.00	10.15 ± 1.99	10.61 ± 1.26	10.69 ± 1.19	12.00 ± 0.00	0.060
*P*-value		0.014		0.923		0.011			0.149			0.112								0.011	0.449
Preventive behaviors	7.55 ± 1.71	6.51 ± 1.86	8.22 ± 1.56	7.05 ± 1.84	7.12 ± 2.41	7.12 ± 1.78	7.02 ± 1.69	7.00 ± 1.87	7.45 ± 2.07	6.92 ± 1.77	8.00 ± 1.41	7.00 ± 2.49	7.44 ± 1.47	6.92 ± 1.75	7.80 ± 3.19	5.77 ± 1.20	6.15 ± 2.51	5.53 ± 1.19	8.00 ± 1.49	6.00 ± 0.00	-0.088
*P*-value		0.000		0.066		1.00			0.439			0.017								0.000	0.266

* Results based on ANOVA with two-way.

** Results based on *t*-test.

## Discussion

The aim of this study was to determine the preventive behaviors against Covid-19 based on the constructs of the HBM in the clients of health centers in Zabol. The mean score of awareness of Covid-19 was higher than other variables and was at a good level. This finding was consistent with the results of a study by Jose *et al*. in 2020 (Jose *et al*., 2021). Considering that a large amount of training in Iran in the field of Covid-19 has been provided through the mass media of the Radio and Television, the personnel of the Ministry of Health, cyberspace, the distribution of warning banners and training campaigns, this widespread information seems to have played an important role in raising awareness of Covid-19 so that according to the participants, the most important source of information was the Ministry of Health, television, and social networks. The results of the study by Shilpa *et al.* also showed that in order to prevent the flu, most people used mass media such as radio and television. Therefore, due to the growing importance of mass media and social networks and due to the importance of non-aggregation in order to reduce the transmission of Covid-19, it is possible to use the high potential and capacity of mass and social media for education, increasing awareness and changing people’s behavior towards preventive behaviors against Covid-19.

More than half of the study population had good levels of Covid-19 prevention behaviors. This finding is consistent with the results of the study by Taghrir as the results of this study showed that only 5.8% of medical students scored low on preventive behaviors. A study in Myanmar found that only 22% of the public’s preventive behavior was acceptable, with 45% of participants washing their hands regularly, and 47% of them always covered their mouth and nose while sneezing or coughing and only 34% refused to travel. In this study, the reason for the low level of preventive behaviors in Myanmar compared to other countries was the insufficient public awareness of coronavirus in this country (Mya *et al*., 2020).

According to the results of the present study, the use of masks when leaving home is more observed than other behaviors. A study conducted in 2006 of the influenza epidemic found that people’s perceptions of the effectiveness of preventive behavior in preventing disease play a key role in observing preventive behaviors (Rubin *et al.*, 2009).

The mean perceived sensitivity score of most of the study population was at a good level, which was in line with the results of the study by Clark *et al*. (2020). High perceived sensitivity of individuals indicates that they believe that they are at high risk of developing the disease. Therefore, a person’s perception of the risk of exposure to Covid-19 may lead to favorable preventive behaviors in the study population. On the other hand, based on the findings, the mean perceived score of most of the subjects was at a good level. This finding was consistent with the results of studies by Jose *et al.* and Li *et al.* in 2020. The results of a study in China showed that high perceived severity increased negative emotions, cell phone use, and preventive behaviors against Covid-19 (Li *et al.*, 2020).

In the present study, the mean score of perceived benefits in the majority of individuals was at a good level. This finding was consistent with the results of studies by Raamkumar *et al*. and Jose *et al*. in 2020. In these studies, the mean score of perceived benefits in the subjects showed a high understanding of the subjects of benefits of performing preventive behaviors against Covid-19. The more people have awareness and understanding of the benefits of preventive measures, the better prepared they are mentally to do these activities and the more likely they are to adopt the given behaviors (Akter, 2021). It seems that the level of awareness and information about this disease has changed the attitude of the study population towards the benefits of preventing behaviors against Covid-19.

Based on the results of this study, perceived barriers of individuals to perform preventive behaviors against Covid-19 were at a high level. The results of the studies showed that the perceived barriers of the subjects were low (Raamkumar *et al*., 2020; Jose *et al*., 2021). The results of a study in Australia showed that about 33% of people believed that their health was seriously affected during a pandemic (Seale *et al*., 2009). In a study in Iran in 2020, it was shown that the perceived barriers of individuals to perform preventive behaviors against Covid-19 were moderate (Khazaee-Pool *et al*., 2020). This indicates that the subjects face more barriers and problems in adopting preventive behaviors. In addition, the objective and psychological costs of the recommended activities were high. Therefore, health policymakers and managers must take action to reduce behavioral barriers as much as possible through a series of interventions and programs.

In this study, the perceived self-efficacy dimension was the strongest construct in predicting the prevention behaviors against Covid-19. The more people feel motivated, capable and hoping to succeed in fighting the coronavirus, the more individual health behaviors show and vice versa. Self-efficacy is a person’s belief in his or her ability to perform behavior (Jeihooni & Rakhshani, 2019). Individuals’ behavior largely depends on the complexity and difficulty of an activity (Bates *et al.*, 2020). In this study, self-efficacy refers to the extent to which a person feels that they can use protective and preventive measures to combat the coronavirus. In other words, it was the level of motivation and ability of people to continue to observe healthy behaviors against the outbreak of coronavirus. In fact, the simpler and more feasible the implementation of preventive behaviors, the more the implementation of preventive behaviors will increase. This finding was consistent with the results of studies by Khazaee-Pool 2020, Bates *et al*. and Jeihooni and Rakhshani (2019). Therefore, using self-efficacy techniques such as increasing people’s awareness of their abilities, verbal encouragement, and providing appropriate role models can play an important role in promoting preventive behaviors against Covid-19.

The results showed that there is a positive and significant correlation between awareness and perceived barriers. People who were more aware of the disease and its complications were more sensitive. The results of correlation coefficient showed that preventive behaviors have a positive and significant correlation with perceived benefits and self-efficacy. The degree of correlations was poor and moderate, but among them, the strongest correlation was observed between perceived barriers and benefits. People who were more aware of the benefits of taking Covid-19 preventative behaviors were more likely to adopt Covid-19 preventive behaviors. Based on the results of regression analysis, the constructs of the HBM were able to predict a total of 36% of the variance of the preventive behavior against Covid-19. Therefore, these findings weakly showed the application of the HBM in predicting the preventive behavior against Covid-19. However, given that the perceived self-efficacy construct was the strongest predictor of Covid-19 preventive behavior, appropriate interventions could be designed to increase perceived self-efficacy to improve Covid-19 preventive behaviors. One of the limitations of this study is the collection of questionnaire information in a self-report manner. In addition, the cross-sectional nature of the study was another limitation that intervention studies in this area are recommended.

## Conclusion

Since no specific and definitive treatment for corona has been found yet, taking preventive measures can be the best way to prevent the spread of this disease in the community. Community participation in disease prevention and control programs, for example, raising awareness to prevent and reduce COVID-19 infection cases, requires the cooperation of all people. Sending informatory text messages, training health personnel in schools and health center staff, arranging educational cartoon TV shows and animations for children, implementing virtual clinics, and providing educational brochures, pamphlets, and booklets help us prevent and control the disease.
